# Special Issue “Hydrogen Storage and Fuel Cells: Materials, Characterization and Applications”

**DOI:** 10.3390/ma15020423

**Published:** 2022-01-06

**Authors:** Rolando Pedicini

**Affiliations:** National Research Council, Institute for Advanced Energy Technologies “Nicola Giordano” (CNR-ITAE), via Santa Lucia Sopra Contesse, 5, 98126 Messina, Italy; rolando.pedicini@itae.cnr.it

Hydrogen is a green energy vector that is considered to be one of the most promising fuels for the future. One of the devices that uses hydrogen as fuel is the fuel cell. Through this electrochemical system, it is possible to produce clean electricity in which the waste product is steam water. There are different types of fuel cells, but in this Special Issue we will only consider devices that operate at low temperatures and that use hydrogen and/or alcohol as fuel.

Despite the high expectations surrounding hydrogen vehicles, especially for the possibility of increasing the autonomy of electromobility, the production of fuel cells is still a costly process today. Their high costs are due in part to the used materials, such as platinum-based catalysts and Nafion^®^ membranes, and the solutions and production techniques utilized.

A further reduction in costs is linked to the search for new materials; in fact, the world of research is moving towards a cheaper solution for each component of the PEFC-type stacks. The characteristics of these new materials must aim, in addition to reducing costs, also to improve some properties, such as the increase in electrochemical performance in conditions of low humidification of the reagent gases, the mechanical stability of the membranes even at temperatures above 120 °C, no corrosion of components and reduction of overall dimensions.

The high cost of hydrogen-powered systems is also caused by the high cost of producing hydrogen. Only green hydrogen has an overall cost of production of almost zero, but the energy storage systems from photovoltaic and wind energy that supply energy to the electrolysers are still complex systems, even if, in recent years, in many countries, it is spreading quite widely. To improve and facilitate the transport of hydrogen, one of the research activities followed, but still with many question marks, is the use of a liquid organic hydrogen carrier (LOHC). The endothermic dehydrogenation phases followed by the purification of the hydrogen formed are considered the main problems that limit the overall efficiency of the storage cycle; moreover, the irreversibility of the process makes these methods still under study.

This Special Issue collects some of the issues mentioned above in a total of seven original publications and one review. A brief description of these works, which I am honored to edit as a Guest Editor, is given below to highlight the high quality of these researches.

An interesting LOHC study was performed using formic acid as a base material [1]. In particular, a selective production of hydrogen from the catalytic decomposition, using iridium and iridium–palladium nanoparticles, was studied. The percentage of conversion of formic acid to hydrogen is closely related to several experimental parameters, such as temperature, stirrer rate and the initial formic acid concentration. It was found that a Pd load between 1 to 2 wt% and 1 wt% of Ir on activated carbon gives a 43% of conversion after 4 h at room temperature. Increasing the temperature to up to 60 °C further improves the conversion rate to up to 63%. With a decrease in the formic acid concentration from 0.5 to 0.2 M, a conversion yield improvement of 81% was also obtained.

The electrochemical deposition technique of nanocrystalline Pt on carbon-based electrodes was carefully studied, and the research results are reported in [2]. The nucleation mechanism, the variation of the oxidation states and the Pt structure’s growth were extensively studied and analyzed using various characterization techniques, such as X-ray diffraction, X-ray photoelectron spectroscopy and electron microscopy field emission scanning. By varying some synthesis parameters, such as current density, time, temperature, pH during the deposition process, this electrochemical deposition technique allows synthesizing a significant amount of Pt nanoparticles that have the most varied morphologies and shapes, such as sphere, flower, core-flower and a rod-like structure. In addition to the ease of synthesis, this work provides a highly scientific and industrial contribution related to a possible scale-up process of these materials for fuel cell applications.

An interesting contribution regarding the development of materials used as electrodes of electrochemical devices, such as fuel cells, is reported in [3]. The study was carried out on the assumption that the sintering of the stabilized Zr oxide with Y oxide occurs in certain conditions of T and the presence of air rich in O_2_ and Ni (named 8YSZ). The authors randomly verified that the simultaneous presence of Ni and Mo significantly increases the yield and speed of sintering, but, above all, the electrochemical performance in terms of ionic conductivity; the results of the Arrhenius plots suggest a conductivity ionic of 0.2 S/cm at 1000 °C. This ionic conductivity value was slightly higher than the value obtained (0.1 S/cm) under the same experimental conditions. Morphology studies performed using a scanning electron microscope highlighted the spontaneous generation of nickel-molybdenum nano-bars on 8YSZ samples after being left under vacuum in the SEM chamber, suggesting the evaporation of a possible nickel-molybdenum compound from the fracture surfaces of the specimen. Although the reason for this improvement in sintering and electrochemical performance is not currently known, understanding it could lead to the development of other metal combinations that could improve both sintering performance and ion conductivity.

The focus of the following manuscripts is related to the so-called “heart” component of the fuel cell, the polymeric membrane.

A study conducted by D. Julius et al. [4] proposes an innovative technique for preparing a proton exchange membrane (PEM) consisting of an aliphatic copolymer having a hydrophobic and a hydrophilic part. In the hydrophobic part, units of glycidyl methacrylate (GMA) are linked, spaced by segments of acrylonitrile (PAN). The hydrophilic part of this block copolymer comprises the potassium-based salt (SPM) 3-sulfopropyl methacrylate. This copolymer was synthesized through one-pot radical polymerization (ATRP). The polymeric membrane was obtained by pouring the solution into which an organic diamine, ethylenediamine (EDA) was introduced to crosslink the chains of the block copolymer. Consequently, the developed membrane has two phases, in which the hydrophobic domains are agglomerated or bridged by the bonds derived from EDA, while the hydrophilic domains constitute the primary conduction channels of the proton. The hydrophobic crosslinking block in situ using a hydrophilic crosslinking agent represents the merit aspect as it leads to improved proton conductivity and dimensional stability in the alcohol fuel (methanol or ethanol). The characterizations performed, both electrochemical and physico-chemical, were compared with the commercial membrane Nafion^®^ 117. The PEM with the optimized composition demonstrates slightly better fuel cell performance than Nafion 117. Finally, this di-block ionomer is not fluorinated and, therefore, it favors the reduction of the costs of materials and the environment.

In addition to the synthesis and the materials chosen for the membrane’s development, their treatment is quite important as it can lead to evident changes in their chemical-physical characteristics and, in particular, in the fuel cell performances. The work reported below [5] reports an interesting research activity of such pre-treatments on a commercial membrane, Nafion™ NR212. This membrane was subjected to chemical treatment with HNO_3_ or H_2_O_2_, and the results obtained from the physico-chemical and electrochemical characterizations were compared to NR212 ones as it is not treated. This study showed how the pre-treatment increases the hydrophilicity and the number of water molecules that the sulphonic groups can coordinate; consequently, the swelling also increases, an essential characteristic so that there can be the proton passage inside the membrane (a vehicular mechanism). The chemical-physical characterizations (water uptake, dynamo-mechanical measurements, SEM mapping and proton conductivity and fuel cell tests) performed on the treated and untreated membranes verified that the former show better mechanical stability, better performances in fuel cell and longer life during fuel cell tests.

Another type of membrane that is quite important and particularly studied in the literature is the class of composite membranes. In this work, the authors describe the synthesis methods and the chemical-physical and electrochemical characterizations performed on a phosphosilicate-based membrane with the formula 30P_2_O_5_-70SiO_2_, doped with poly (ethylene oxide) (PEO) and poly (vinyl alcohol) (PVA) using Ti oxide as an additive [6]. The synthesized samples were carefully characterized through Fourier transformed infrared spectroscopy (FTIR), FT-Raman spectroscopy; porosimetry and microstructure techniques through BET measurements; mechanical properties through dynamo-mechanical measurements; thermal analysis through differential thermal analysis (DTA) and thermogravimetry (TG); electrical and electrochemical measurements through electrical impedance spectroscopy (EIS) to alternating current measurements. Electrical conductivity measurements were performed in different humidity conditions. The values obtained ranged from 10^−4^ to 10^−9^ S/cm for the dehydrated samples, while for the “wet” samples, the values were ~10^−3^ S/cm. The added Ti oxide also modified the pore distribution and the specific surface of the modified glass systems. Based on the analysis of the dispersion of the dielectric losses, it was found that the composite samples show mixed proton mobility with relative contributions to the mass of the material than at the surface of the pore space.

A fairly complete study on a type of polymeric membrane, an alternative to the traditional Nafion^®^, based on composite sulfonated Polyetheretheretherketone (SPEEK) with mesoporous silica [7], was performed. These composite membranes, the amount of oxide ranged from 5 wt% to 15 wt%, have been extensively characterized chemically, physically and electrochemically in a fuel cell, using both hydrogen and methanol as fuel, and in an electrochemical hydrogen compressor. The standard requirements for all applications are high proton conductivity, thermomechanical stability and impermeability to fuel and oxidants. In direct hydrogen fuel cells, the best compromise proved to be the sample containing 5 wt%, whose performance is the best in terms of proton conductivity and stability over time; with the increase in the oxide load, its barrier becomes counterproductive for the proton conductivity. By varying the type of fuel, passing from hydrogen to methanol, the insertion of the inorganic, in addition to acting as a barrier, causes greater porosity, which can be seen in the high cross-over value obtained in the sample with 15 wt% of oxide. The best compromise was 10 wt%. In the case of the electrochemical compressor, the inclusion of inorganic materials led to a backscatter, limiting the compression capacity, so in this case, the starting material, SPEEK, shows the best performance.

As mentioned earlier, the polymeric membrane is the critical component of a polymer electrolyte fuel cell. The lifespan of a PEM is the main factor limiting the commercialization of PEMFC [8]. Under the working conditions of vehicles powered by fuel cells, the output power often has to change to meet the vehicle’s energy demand. This condition determines that the PEM will undergo frequent dynamic changes in temperature, humidity, reactant demand, current and potential, which will accelerate the mechanical and chemical degradation of the PEM. In order to fully understand the behavior of a membrane when a fuel cell is in operation, this review provides handy information on the mechanical and chemical degradation behavior and its causes, as well as mitigation strategies, in order to provide a possible preferential direction of fragility for PEM design and control strategy of the fuel cell system. It should be noted that the synthesis parameters of a PEM, such as thickness membrane, the equivalent weight value of the polymer, the composition and the content of possible additives must be considered in a design study of a PEMFC. Parallel to what has been said, the study and development of materials for the entire fuel cell system, such as electrodes, bipolar plates, etc., is significant to optimize the entire fuel cell and make it more resistant and lasting over time.
